# The YTHDC1/GLUT3/RNF183 axis forms a positive feedback loop that modulates glucose metabolism and bladder cancer progression

**DOI:** 10.1038/s12276-023-00997-z

**Published:** 2023-06-01

**Authors:** Bin Yan, Xurui Li, Mou Peng, Yali Zuo, Yinhuai Wang, Pian Liu, Weigang Ren, Xin Jin

**Affiliations:** 1 Department of Urology, The Second Xiangya Hospital, Central South University, 410011 Changsha, Hunan China; 2 Uro-Oncology Institute of Central South University, 410011 Changsha, Hunan China; 3 Cancer Center, Union Hospital, Tongji Medical College, Huazhong University of Science and Technology, 430022 Wuhan, China; 4 Department of Urology, Hunan Provincial People’s Hospital, The First Affiliated Hospital of Hunan Normal University, 410005 Changsha, Hunan China

**Keywords:** Bladder cancer, Cancer metabolism

## Abstract

Aberrant glucose metabolism is a characteristic of bladder cancer. Hyperglycemia contributes to the development and progression of bladder cancer. However, the underlying mechanism by which hyperglycemia promotes the aggressiveness of cancers, especially bladder cancer, is still incompletely understood. N6-methyladenosine (m^6^A) modification is a kind of methylation modification occurring at the N6 position of adenosine that is important for the pathogenesis of urological tumors. Recently, it was found that the m^6^A reader YTHDC1 is regulated by high-glucose conditions. In our study, we revealed that YTHDC1 is not only regulated by high-glucose conditions but is also downregulated in bladder cancer tissue and associated with the prognosis of cancer. We also showed that YTHDC1 suppresses the malignant progression of and the glycolytic process in bladder cancer cells in an m^6^A-dependent manner and determined that this effect is partially mediated by GLUT3. Moreover, GLUT3 was found to destabilize YTHDC1 by upregulating RNF183 expression. In summary, we identified a novel YTHDC1/GLUT3/RNF183 feedback loop that regulates disease progression and glucose metabolism in bladder cancer. Collectively, this study provides new insight regarding the pathogenesis of bladder cancer under hyperglycemic conditions and might reveal ideal candidates for the development of drugs for bladder cancer.

## Bladder cancer: How sugar supply promotes tumor growth

Understanding the links between glucose metabolism and bladder cancer (BC) may help find novel therapies. High blood sugar and cancer are linked—abundant energy fuels rapid tumor growth—but the mechanisms are not completely understood, especially in BC. Bin Yan at Central South University, Changsha, China, and co-workers investigated how YTHDC1, a protein known to be suppressed by high blood sugar, affects BC. They found that BC tissues had low levels of YTHDC1 which was associated with a poor prognosis. Boosting YTHDC1 levels slowed tumor cell growth by decreasing levels of GLUT3, a glucose transporter that accelerates sugar input to cancer cells. Further investigation revealed a positive feedback loop between the two proteins, because GLUT3 tags YTHDC1 for degradation. These results illuminate how high blood sugar promotes BC growth and suggest possible drug development targets.

## Introduction

Bladder cancer is the most common malignancy of the urinary system^1,2^. Bladder cancer is the fifth most common cancer^3^, and nearly 170,000 patients die of this disease worldwide each year^3–5^. For muscle-invasive and advanced bladder cancer, platinum-based chemotherapy has been considered the standard systemic therapy over the past 40 years^5^. Due to the high mutation burden of bladder cancer, immunotherapy has been used to treat bladder cancer and has substantially improved the prognosis of patients in recent years^6^. However, studies exploring the molecular mechanism of advanced bladder cancer are also needed for the development of new therapeutic strategies.

Aberrant glucose metabolism is one of the characteristics of malignant tumors^7^. In 1885, it was first reported that patients with cancer exhibited high blood glucose concentrations. Later, Warburg et al. noted that cancer tissues maintained a higher glucose utilization rate than normal tissues^8^. High blood glucose, or hyperglycemia, is usually caused by stress, diabetes mellitus, inflammation, or some kinds of drugs^9–11^. Among these causes, diabetes mellitus is the most common reason for long-term elevation of the plasma glucose concentration. Large cohort studies have demonstrated that diabetes mellitus-induced hyperglycemia is crucial for the tumorigenesis of solid tumors, such as liver^12^, pancreatic^13^, and bladder^14,15^ cancers. Lattermann et al. analyzed glucose metabolism in patients with bladder cancer. They found that the plasma concentration of glucose was elevated in patients with bladder cancer due to the lower rate of glucose clearance^16^. Moreover, it has been mentioned that hyperglycemia contributes to the development and progression of bladder cancer in diabetic patients. High glucose-mediated promotion of metastasis leads to death in patients with bladder cancer and diabetes^17^. Mechanistically, the excessive glucose supply available to cancer cells and the high glycolysis rate in those cells provides a suitable microenvironment for the development and progression of malignant tumors^18^. However, the underlying mechanism by which hyperglycemia promotes the aggressiveness of cancers, especially bladder cancer, is still incompletely understood.

N6-methyladenosine (m^6^A) modification is a kind of methylation modification occurring at the N6 position of adenosine that is a conserved transcriptional modification in eukaryotic messenger RNA (mRNA)^19,20^. A number of studies have demonstrated that m^6^A modification is important for the pathogenesis of urological tumors^21^. m^6^A is installed by m^6^A methyltransferases, removed by demethylases and recognized by m^6^A-binding proteins^20^. m^6^A-binding proteins are modification readers and include YTHDC1/2, IGF2BP1/2/3, YTHDF1/2/3 and so on^20^. Each m^6^A modification reader performs distinct functions in cancer cells^22^. Many studies have reported that m^6^A modification is closely associated with glucose metabolism^23–25^. Recently, Liang et al. found that the m^6^A reader YTHDC1 is regulated by high-glucose conditions^26^. In our study, we found that YTHDC1 is not only regulated by high-glucose conditions but also downregulated in bladder cancer tissue and associated with the prognosis of cancer. Then, we showed that YTHDC1 participates in regulating glucose metabolism and that this effect might be mediated by GLUT3 downregulation in bladder cancer cells. On the other hand, we found that GLUT3 destabilizes YTHDC1 by upregulating RNF183 expression. Thus, we identified a novel YTHDC1/GLUT3/RNF183 feedback loop in bladder cancer cells.

## Materials and methods

### Cell lines and reagents

The bladder cancer cell lines 5637 and T24 were purchased from Yuchi Biology (Shanghai, China) with authentication by short tandem repeat (STR) profiling as previously reported^27^. T24 cells were cultured in RPMI-1640 medium (Gibco, Grand Island, NY, USA) supplemented with 10% fetal bovine serum (FBS; AC03L055, Shanghai Life-iLab Biotech, China) and 1% penicillin. 5637 cells were cultured in Dulbecco’s modified Eagle’s medium (DMEM; Gibco, USA) supplemented with 10% FBS and 1% penicillin. All cells were maintained in an incubator at 37 °C in 5% CO_2_. Lipofectamine 2000 was obtained from Thermo Scientific (Shanghai, China). Glucose (# S3131) and mannitol (# S2381) were purchased from Selleck (Shanghai, China).

### Constructs and transfection

The HA-YTHDC1, HA-GLUT3, and Flag-RNF183 plasmids were constructed by inserting the cDNA of YTHDC1, GLUT3, and RNF183, respectively, into OmicsLinkTM Expression Clone (CMV Promoter) vectors (GeneCopoeia, Guangzhou, China). The siRNAs targeting YTHDC1 and RNF183 were purchased from RiboBio (Guangzhou, China). The short hairpin RNAs (shRNAs) were obtained from GeneCopoeia (Guangzhou, China). The plasmids and siRNAs were transfected into cells by using Lipofectamine 2000 following the manufacturer’s instructions. The shRNAs were transfected into the cells by using Lipofectamine 2000. After puromycin selection, we obtained cells stably transfected with the shRNAs. The sequences of the shRNAs and siRNAs are listed in Supplementary Table 1.

### Immunoprecipitation, glutathione S-transferase pulldown assay, and western blot analysis

The details of the immunoprecipitation and western blotting procedures were described previously^27^. In brief, the protein was harvested from cells with 1× RIPA buffer (Beyotime Biotechnology, China). The protein concentration in the cell lysate was evaluated by an Enhanced BCA Protein Assay Kit (P0010S, Beyotime Biotechnology, China). For immunoprecipitation (IP), protein from the bladder cancer cells was incubated with IgG or a specific primary antibody and protein A + G beads (#P2029, Beyotime, China) for 12 h at 4 °C. Then, the beads were washed with 1× RIPA buffer 6 times on the ice. The beads were boiled with loading buffer and subjected to western blot analysis.

For the glutathione S-transferase (GST) pulldown assay, GST-tagged plasmids were transformed into *E. coli* BL21 cells. GST fusion proteins were immobilized on BeyoMag™ Anti-GST Magnetic Beads (P2138, Beyotime, Shanghai, China). The GST beads were cocultured with the cell lysate for 4 hours. Then, the beads were washed with 1× RIPA buffer 6 times on the ice. The beads were boiled with loading buffer and subjected to western blot analysis.

For western blot analysis, proteins were separated by SDS‒PAGE and immunoblotted with primary and secondary antibodies. The antibodies used were as follows: anti-YTHDC1 (#29441-1-AP, Proteintech, 1:500 dilution), anti-GLUT3 (#20403-1-AP, Proteintech, 1:1000 dilution), anti-RNF183 (#ab197321, Abcam, 1:1000 dilution) and anti-beta Actin (#66009-1-Ig, Proteintech, 1:5000 dilution).

### Immunohistochemical (IHC) staining

Tissue microarray slides (#U100Bl01) were purchased from Bioaitech, China. An anti-YTHDC1 antibody (#29441-1-AP, Proteintech, 1:1000 dilution) was used to stain the tissue microarray slide. The method for scoring the staining intensity was described previously^28^.

### Quantitative reverse transcription PCR (RT‒qPCR) and RNA-seq analysis

Total RNA was extracted by using a TRIzol reagent (Thermo Fisher Scientific, USA). RT‒qPCR was performed by using a reverse transcription kit and a PCR kit (#RR037A, PrimeScript™ RT Reagent Kit; #RR430A, TB Green™ Fast qPCR Mix; Takara Bio Inc., Kusatsu, Shiga, Japan) following the manufacturer’s instructions. The primer sequences used for RT‒PCR are listed in Supplementary Table 2. To evaluate RNA stability, bladder cells were treated with actinomycin D (5 μg/mL). Then, the cells were collected at different time points. Total RNA was extracted and analyzed by RT‒qPCR. The mRNA expression level in each group was normalized to that of β-actin.

For RNA-seq analysis, 5637 cells were transfected with the indicated siRNAs. Forty-eight hours post-transfection, cells were harvested and sent to Novogene (Beijing, China) for RNA-seq analysis. Briefly, total RNA was assessed using an RNA Nano 6000 Assay Kit with a Bioanalyzer 2100 system (Agilent Technologies, CA, USA). First-strand cDNA was synthesized using random hexamer primers and M-MuLV Reverse Transcriptase, and then RNase H was used to degrade the RNA. Second-strand cDNA synthesis was subsequently performed using DNA Polymerase I and dNTP. The library fragments were purified with the AMPure XP system (Beckman Coulter, Beverly, USA). Then, the library was sequenced on the Illumina NovaSeq 6000 platform, and featureCounts (v1.5.0-p3) was used to count the reads mapped to each gene.

### RNA immunoprecipitation (RIP) and methylated RNA immunoprecipitation (MeRIP)-qPCR

Whole-cell lysate was obtained by lysing cells with 1× RIPA buffer (Beyotime Biotechnology, China). Ten percent of the lysate was collected and used as input. Then, a primary antibody or IgG and protein A + G beads (#P2029, Beyotime, China) were added to the rest of the cell lysate and incubated for 12 hours at 4 °C. The beads were washed and resuspended in protein kinase K buffer and were then incubated at 55 °C for 30 min with shaking in RNAiso Plus (Takara, 9109). RT‒qPCR was performed by using a reverse transcription kit and a PCR kit (#RR037A, PrimeScript™ RT Reagent Kit; #RR430A, TB Green™ Fast qPCR Mix; Takara Bio Inc., Shingo, Japan) following the manufacturer’s instructions. The primer sequences are listed in Supplementary Table 3. The Magna m^6^A MeRIP Kit (# A-17-10499, A&D Technology, Beijing, China) was used to perform MeRIP-qPCR following the manufacturer’s protocol. The primer sequences are listed in Supplementary Table 4.

### In vitro and in vivo cell proliferation assays

An in vitro cell proliferation assay was carried out following the manufacturer’s instructions for the Cell Counting Kit-8 (CCK-8). In brief, bladder cancer cells were plated in 96-well plates with 1 × 10^3^ cells in each well. Then, CCK-8 reagent (#C0037, Beyotime) was added to each well at different time points. The absorbance at 450 nm was measured and used to quantify the cell proliferation ability.

All animal experiments were approved by the ethics committee of the Second Xiangya Hospital, Central South University (animal license number: 2022588). BALB/c nude mice were obtained from Shulaobao Biotech (Wuhan, China). Bladder cancer cells transduced with the indicated shRNAs were subcutaneously injected into the left dorsal flanks of the mice (1 × 10^7^ cells per mouse). Tumor volume was calculated using the formula (*L* × *W*^2^)/2. Once the mice were euthanized, the tumors were excised and weighed.

### Statistical analysis

The experimental data are presented as the mean ± standard error of the mean (mean ± SEM) values. The sample size (*n*) for each statistical analysis is provided in the figure legends. GraphPad Prism 5 software was used to calculate *P* values using unpaired two-tailed Student’s *t*-test to compare values between two groups or one-way analysis of variance (ANOVA) followed by Tukey’s post hoc test for multiple comparisons to compare values among more than two groups. Differences were considered statistically significant when the *P* value was less than 0.05. In all cases, the significance of differences is indicated as follows: *P < 0.05; **P < 0.01; ***P < 0.001; not significant (ns), *P* > 0.05.

## Results

### Glucose-associated YTHDC1 is abnormally downregulated in bladder cancer

**Fig. 1:**
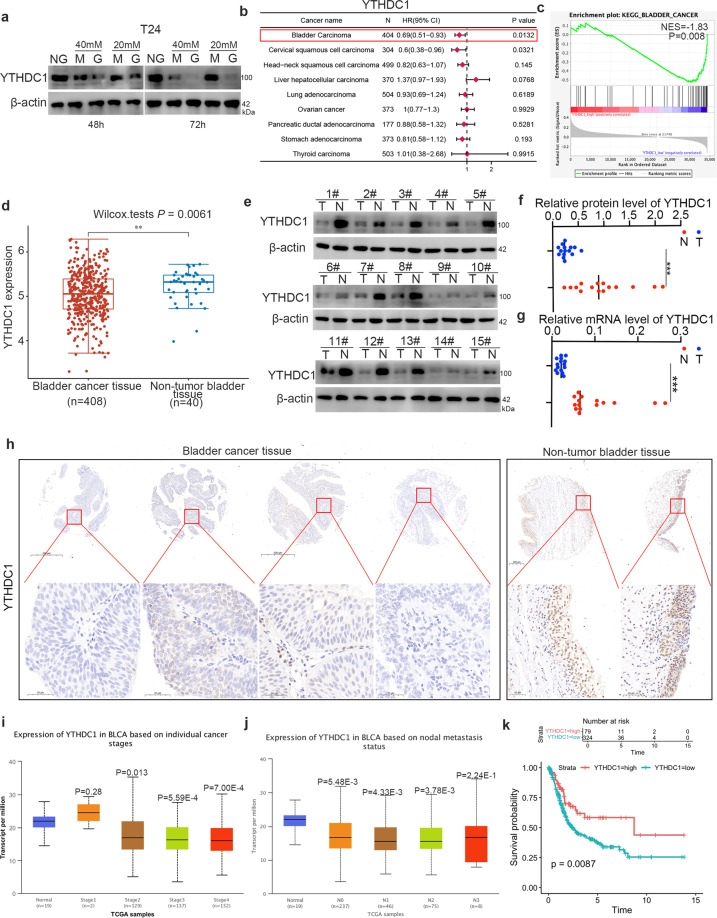
Glucose-related YTHDC1 is abnormally downregulated in bladder cancer. **a** T24 cells were treated with the indicated chemicals for 48 or 72 h. Then, the cells were harvested for western blot analysis. NG indicates normal glucose. M indicates mannitol, which was used to generate hypertonic conditions similar to those generated by high glucose but could not be used by cells. G indicates glucose. **b** The ENCORI web tool was applied to analyze the expression level of YTHDC1 in different types of malignancies. The *P* values and HRs are indicated. **c** GSEA of the TCGA dataset showed that YTHDC1 is negatively correlated with pathways associated with bladder cancer. **d** The expression of YTHDC1 in the TCGA-BLCA dataset was analyzed. *P* values are indicated. **e–g** The protein and mRNA levels of YTHDC1 in bladder cancer specimens and adjacent normal tissues were analyzed by western blotting (**e**) and RT‒qPCR (**g**). The protein level of YTHDC1 was quantified by ImageJ software (**f**). Student’s *t*-test was applied to compare values between the two groups. *N* = 15, ***P < 0.001. **h** The bladder cancer tissue microarray was analyzed by IHC staining by using the anti-YTHDC1 antibody. **i** and **j** The expression of YTHDC1 in samples of different stages (**i**) and nodal metastasis statuses (**j**) in the TCGA-BLCA dataset was analyzed by using the UALCAN web tool (http://ualcan.path.uab.edu/). *P* values are indicated in the panel. **k** Overall survival of patients with bladder cancer in the low and high YTHDC1 expression groups. *P* values are indicated in the figure.

Since both hyperglycemia and m^6^A modification are important for the development of malignant tumors^19,29^, we aimed to further explore the connection between hyperglycemia and m^6^A modification in bladder cancer. Recently, a study reported that YTHDC1 was downregulated in high glucose-treated keratinocytes^26^. Similarly, we showed that hyperglycemia induced by treatment with glucose (G) but not hypertonic conditions (mimicked by treatment with mannitol (M)) resulted in downregulation of YTHDC1 protein expression in T24 cells (Fig. 1a). We also found that hyperglycemia induced by treatment with glucose but not hypertonic conditions did not change the mRNA level of YTHDC1 in T24 cells (Supplementary Fig. 1a). Notably, TCGA dataset analysis indicated that the hazard ratio of the expression of YTHDC1 was less than 1.00 in bladder cancer (*P* = 0.0132) (Fig. 1b). Gene set enrichment analysis (GSEA) was performed after dividing the bladder cancer specimens from the TCGA dataset into the high YTHDC1 expression group and low YTHDC1 expression group, and it showed that YTHDC1 was negatively correlated with the KEGG_BLADDER_CANCER signaling pathway (*P* = 0.008, NES = −1.83) (Fig. 1c). Then, we found that YTHDC1 was significantly downregulated in bladder cancer tissues compared with nontumor tissues in the TCGA dataset, in specimens from our hospital and in a bladder cancer tissue microarray obtained from Bioaitech (#U100Bl01) (Fig. 1d–h). Furthermore, analysis of the UALCAN dataset (http://ualcan.path.uab.edu/) demonstrated that YTHDC1 expression was also decreased in different stages of bladder cancer and in patients with lymph node metastasis (Fig. 1i and j). Finally, we showed that low expression of YTHDC1 was significantly correlated with an unfavorable prognosis in patients with bladder cancer (Fig. 1k). Together, these data indicate that YTHDC1 is associated with the prognosis of patients with bladder cancer.

### YTHDC1 is responsible for suppressing the aggressive behaviors of bladder cancer cells

**Fig. 2:**
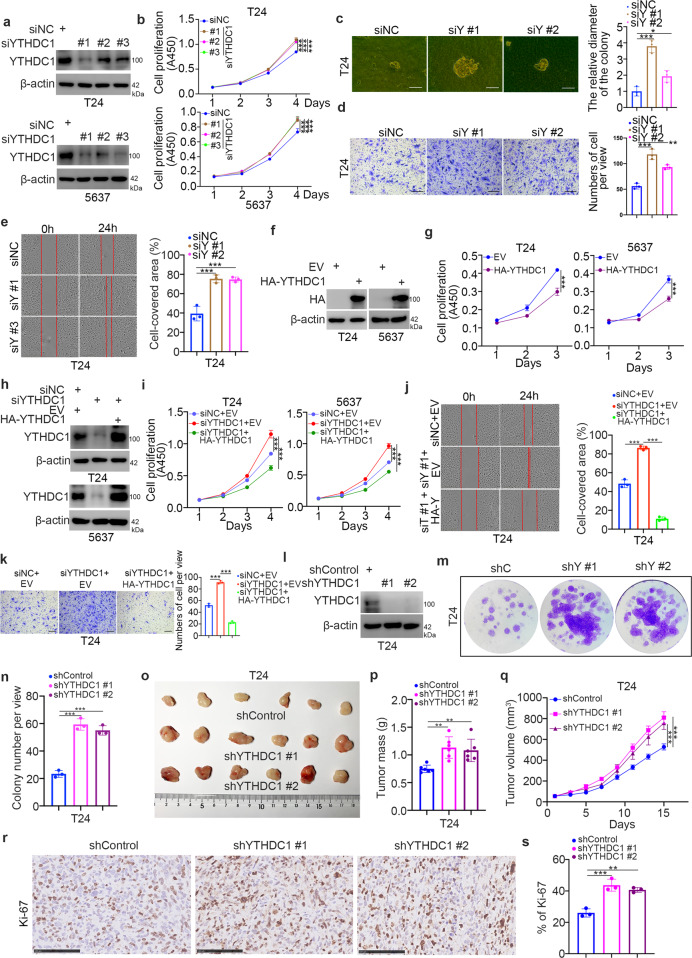
YTHDC1 suppresses the malignant progression of bladder cancer cells. **a–e** T24 and 5637 cells were transfected with the indicated siRNAs for 48 h. Cells were collected for western blot analysis (**a**), a CCK-8 assay (**b**), a colony formation assay (**c**), a transwell assay (**d**), and a wound healing assay (**e**). The data are presented as the mean ± SEM of three replicates. *P < 0.05; **P < 0.01; ***P < 0.001. **f** and **g** T24 and 5637 cells were transfected with the indicated plasmids for 48 h. The cells were harvested for western blot analysis (**f**) and a CCK-8 assay (**g**). The data are presented as the mean ± SEM of three replicates. ***P < 0.001. **h–k** T24 and 5637 cells were transfected with the indicated constructs for 48 h. Cells were collected for western blot analysis (**h**), a CCK-8 assay (**i**), a wound healing assay (**j**), and a transwell assay (**k**). The data are presented as the mean ± SEM of three replicates. ***P < 0.001. **l–m** T24 cells were infected with the indicated shRNAs for 72 h. After puromycin selection, cells were collected for western blot analysis (**l**), a colony formation assay (**m** and **n**), and a nude mouse xenograft assay (**o**–**s**). For the colony formation assay and Ki-67 IHC staining, data are presented as the mean ± SEM of three replicates. For the xenograft assay, data are presented as the mean ± SEM of six replicates. **P < 0.01; ***P < 0.001.

The above findings indicated a strong link between YTHDC1 and bladder cancer, but the biological role of YTHDC1 in bladder cancer is still an enigma. First, YTHDC1 was silenced in T24 and 5637 cells by transfection with gene-specific siRNAs (Fig. 2a). The results of CCK-8 and colony formation assays indicated that knockdown of YTHDC1 increased the proliferation of bladder cancer cells (Fig. 2b and c). Moreover, silencing YTHDC1 enhanced the invasion and migration capabilities of T24 cells (Fig. 2d and e). In contrast, YTHDC1 was overexpressed in both T24 and 5637 cells by transfection of plasmids expressing HA-tagged YTHDC1 (Fig. 2f). We found that YTHDC1 overexpression inhibited the proliferation of bladder cancer cells (Fig. 2g). Moreover, rescuing the expression of YTHDC1 attenuated the proliferation-, migration-, and invasion-promoting effects induced by knockdown of YTHDC1 in bladder cancer cells (Fig. 2h–k). In addition, the results of the colony formation assay and nude mouse xenograft assay showed that YTHDC1 depletion enhanced bladder cancer cell growth in vitro and bladder tumor growth in vivo (Fig. 2l–s). Thus, our results suggest that the downregulation of YTHDC1 contributes to the aggressive behaviors of bladder cancer cells.

### YTHDC1 modulates glucose metabolism and suppresses GLUT3 expression in bladder cancer cells

**Fig. 3:**
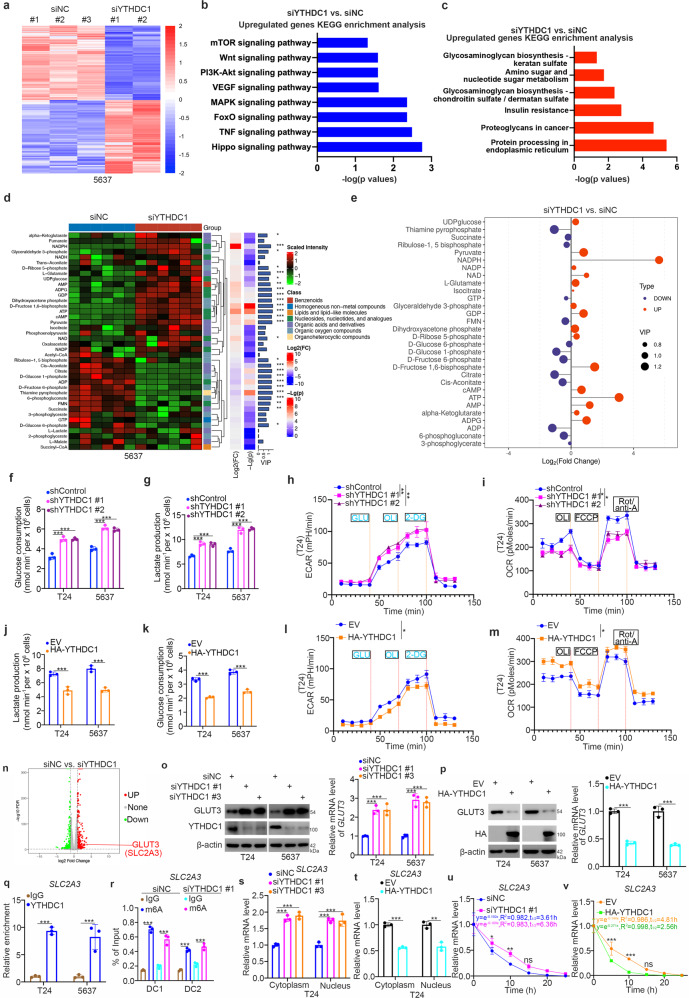
YTHDC1 regulates glucose metabolism and downregulates GLUT3 expression in bladder cancer cells. **a–c** 5637 cells were transfected with the indicated siRNAs for 48 h. Cells were collected for RNA-seq analysis. Then, KEGG enrichment analysis was used to identify the pathways associated with YTHDC1 in bladder cancer cells. **d** and **e** 5637 cells were transfected with the indicated siRNAs for 48 h. Cells were collected for targeted metabolomics related to glucose metabolism. **f–i** T24 and 5637 cells were transfected with the indicated siRNAs for 48 h. Cells and spent medium were collected to measure glucose consumption (**f**), lactate production (**g**), the ECAR (**h**), and the OCR (**i**). The data are presented as the mean ± SEM of three replicates. *P < 0.05; **P < 0.01; ***P < 0.001. **j–m** T24 and 5637 cells were transfected with the indicated plasmids for 24 h. Cells and spent medium were collected to measure lactate production (**j**), glucose consumption (**k**), the ECAR (**l**), and the OCR (**m**). The data are presented as the mean ± SEM of three replicates. *P < 0.05; ***P < 0.001. **n** RNA-seq analysis after knocking down YTHDC1 in 5637 cells. **o** T24 and 5637 cells were transfected with the indicated siRNAs for 48 h. The cells were harvested for western blot analysis and RT‒qPCR. The data are presented as the mean ± SEM of three replicates. ***P < 0.001. **p** T24 and 5637 cells were transfected with the indicated plasmids for 24 h. The cells were harvested for western blot analysis and RT‒qPCR. The data are presented as the mean ± SEM of three replicates. ***P < 0.001. **q** RIP-qPCR was performed in T24 and 5637 cells by using IgG or an anti-YTHDC1 antibody. The data are presented as the mean ± SEM of three replicates. ***P < 0.001. **r** MeRIP-qPCR was performed in T24 and 5637 cells. The data are presented as the mean ± SEM of three replicates. ***P < 0.001. **s** T24 cells were transfected with the indicated siRNAs for 48 h. Cells were collected, and RNA was extracted from the cytoplasm and from the nucleus. RT‒qPCR was performed. The data are presented as the mean ± SEM of three replicates. ***P < 0.001. **t** T24 cells were transfected with the indicated plasmids for 24 h. Cells were collected, and RNA was extracted from the cytoplasm and from the nucleus. RT‒qPCR was performed. The data are presented as the mean ± SEM of three replicates. **P < 0.01; ***P < 0.001. **u** T24 cells were transfected with the indicated siRNAs for 48 h. Then, the cells were treated with actinomycin D (5 μg/mL). Then, the cells were collected at different time points. Total RNA was extracted and analyzed by RT‒qPCR. mRNA expression in each group was normalized to β-actin. **v** T24 cells were transfected with the indicated plasmids for 24 h. Then, the cells were treated with actinomycin D (5 μg/mL). Then, the cells were collected at different time points. Total RNA was extracted and analyzed by RT‒qPCR. mRNA expression in each group was normalized to β-actin.

Next, we explored the underlying mechanism by which YTHDC1 inhibits the malignant progression of bladder cancer cells. Transcriptome sequencing, or RNA-seq, was performed after knocking down YTHDC1 in 5637 cells (Fig. 3a). KEGG enrichment analysis of the RNA-seq data showed that YTHDC1 silencing upregulated many cancer-related pathways, including the Hippo, TNF, MAPK, VEGF, and PI3K-AKT-mTOR signaling pathways (Fig. 3b). Notably, knockdown of YTHDC1 also activated some glucose metabolism-regulated pathways in bladder cancer cells (Fig. 3c). To further investigate the effect of YTHDC1 on modulating glucose metabolism, targeted metabolomics related to glucose metabolism was performed after YTHDC1 knockdown in 5637 cells (Fig. 3d). The results demonstrated that compared with the control treatment, YTHDC1 silencing activated glucose metabolism and resulted in greatly increased ATP and NADPH production (Fig. 3e). Consistent with these findings, we showed that knockdown of YTHDC1 increased the glucose consumption rate, lactate production rate, and extracellular acidification rate (ECAR) but decreased the oxygen consumption rate (OCR) in bladder cancer cells (Fig. 3f–i, Supplementary Fig. 2a and b). In contrast, overexpression of YTHDC1 inhibited the glycolytic process and decreased the ECAR but increased the OCR in bladder cancer cells (Fig. 3j–m, Supplementary Fig. 2c and d). Furthermore, we reanalyzed the RNA-seq data related to YTHDC1 knockdown and found that SLC2A3 (GLUT3) was one of the genes with the largest change in expression after the knockdown of YTHDC1 (Fig. 3n). Subsequent western blot and RT‒qPCR analyses confirmed that YTHDC1 silencing increased but overexpression of YTHDC1 decreased GLUT3 expression in T24 and 5637 cells (Fig. 3o and p). Then, we studied how YTHDC1 regulates the expression of GLUT3 in bladder cancer cells. First, analysis with the Encyclopedia of RNA Interactomes (ENCORI) (https://starbase.sysu.edu.cn/index.php) web tool demonstrated that there are three binding regions for YTHDC1 in SLC2A3 (Supplementary Fig. 3a). The results of RIP assays with the anti-YTHDC1 antibody showed that YTHDC1 bound to SLC2A3 in both T24 and 5637 cells (Fig. 3q). Then, analysis with the RMBase v2.0 (https://rna.sysu.edu.cn/rmbase/index.php) web tool showed that there were many m^6^A modifications in SLC2A3 (Supplementary Fig. 3b). Notably, the m^6^A modification of SLC2A3 in the 3’-UTR was close to the YTHDC1 binding regions. Next, m^6^A RIP-qPCR showed that SLC2A3 was enriched by IP with anti-m6A antibodies in bladder cancer cells (Fig. 3r and Supplementary Fig. 3c). In addition, we showed that the knockdown of YTHDC1 did not change the enrichment of the 3’-UTR of SLC2A3 by the anti-m^6^A antibody (Fig. 3r and Supplementary Fig. 3c). It has been reported that YTHDC1 is involved in regulating the subcellular translocation or stability of its target RNA transcripts^30,31^. Interestingly, we found that knockdown and overexpression of YTHDC1 increased and decreased, respectively, the expression of SLC2A3 in both the cytoplasm and nucleus of T24 cells (Fig. 3s and t). Then, we showed that the changes in YTHDC1 expression affected the mRNA stability of *SLC2A3* in T24 cells (Fig. 3u and v). In addition, analysis with the ENCORI web tool indicated that the mRNA level of YTHDC1 was negatively correlated with that of GLUT3 in bladder carcinoma, testicular germ cell tumor, sarcoma, and lung squamous cell carcinoma (Supplementary Fig. 3d). The principal function of YTHDC1 is to regulate exon inclusion during the splicing process as an m6A reader^32,33^. To investigate whether YTHDC1 regulates SLC2A3 gene splicing, we first analyzed the Ensembl database (https://grch37-archive.ensembl.org/) and found that the SLC2A3 pre-mRNA can be spliced to generate a short isoform (SLC2A3-010) relative to the full-length isoform (SLC2A3-001) (Supplementary Fig. 3e and f). Then, RT‒PCR and RT‒qPCR analysis showed that overexpression of YTHDC1 decreased the production of the full-length isoform of SLC2A3 but did not change the production of the short splice isoform (SLC2A3-010) in T24 cells (Supplementary Fig. 3g). We also demonstrated that in contrast, inhibition of YTHDC1 increased the production of the full-length isoform but not the short splice isoform of SLC2A3 in T24 cells (Supplementary Fig. 3h). Thus, these data indicated that YTHDC1 did not regulate SLC2A3 pre-mRNA splicing in bladder cancer cells. On the other hand, these results suggest the possibility that YTHDC1 is involved in the exon inclusion process. Taken together, these results demonstrate that YTHDC1 regulates glucose metabolism and suppresses the expression of GLUT3 in bladder cancer cells.

### GLUT3 plays an important role in promoting the malignant progression of bladder cancer cells

**Fig. 4:**
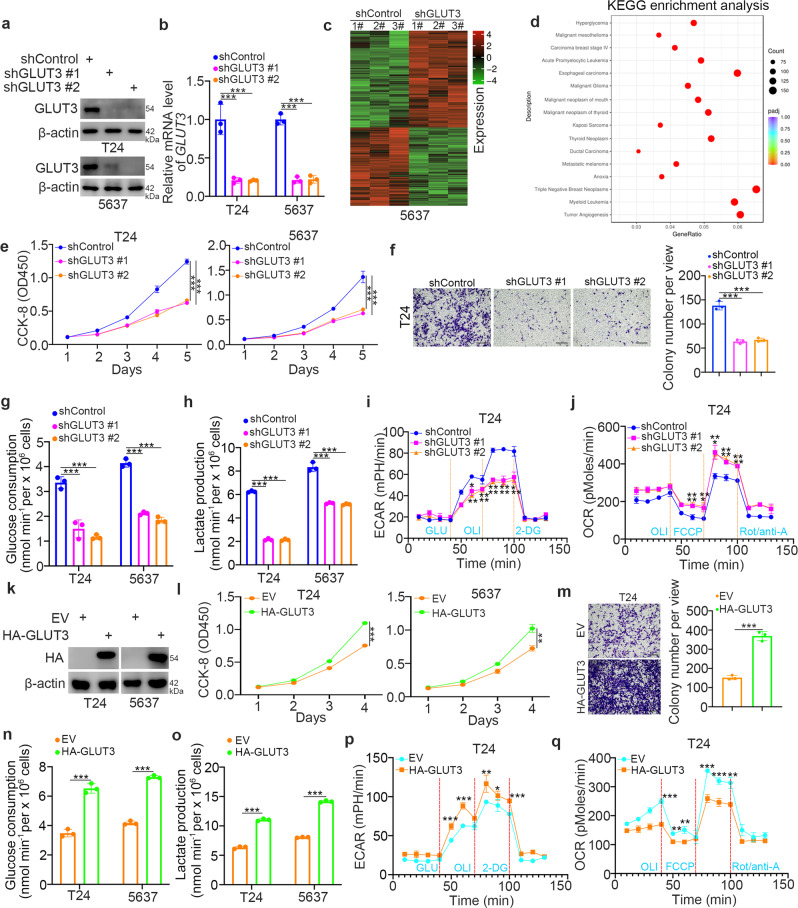
GLUT3 is critical for promoting the progression of bladder cancer. **a** and **b** T24 and 5637 cells were transfected with the indicated shRNAs for 48 h. Cells were collected for western blot analysis (**a**) and RT‒qPCR (**b**). The data are presented as the mean ± SEM of three replicates. ***P < 0.001. **c** and **d** 5637 cells were transfected with the indicated shRNAs for 72 h. After puromycin selection, cells were collected for RNA-seq analysis. KEGG enrichment analysis was performed (**d**). **e–j** T24 and 5637 cells were transfected with the indicated shRNAs for 48 h. Cells were collected for CCK-8 (**e**), transwell (**f**), glucose consumption (**g**), lactate production (**h**), ECAR (**i**) and OCR (**j**) assays. The data are presented as the mean ± SEM of three replicates. *P < 0.05; **P < 0.01; ***P < 0.001. **k–q** T24 and 5637 cells were transfected with the indicated plasmids for 24 h. Cells were collected for western blot analysis (**k**) and for CCK-8 (**l**), transwell (**m**), glucose consumption (**n**), lactate production (**o**), ECAR (**p**), and OCR (**q**) assays. The data are presented as the mean ± SEM of three replicates. *P < 0.05; **P < 0.01; ***P < 0.001.

GLUT3 functions as a glucose transporter to regulate glucose metabolism in cells^34^. Upregulation of GLUT3 is responsible for promoting invasion and metastasis in glioma and breast cancer^35,36^. GLUT3 has also been reported to contribute to bladder carcinogenesis^37^. Here, we intended to further explore the cancer-related role of GLUT3 in bladder cancer cells. First, RNA-seq was performed after GLUT3 knockdown in bladder cancer cells (Fig. 4a–c). KEGG enrichment analysis indicated that GLUT3 was involved in modulating various pathways in cells, including hyperglycemia-, tumor angiogenesis-, and cancer-related signaling pathways (Fig. 4d). Consistent with previous findings, GLUT3 silencing inhibited cell proliferation and invasion activities (Fig. 4e and f). We also showed that GLUT3 knockdown decreased glucose consumption, lactate production, and the ECAR in T24 and 5637 cells (Fig. 4g–i, Supplementary Fig. 4a–c). We further found that GLUT3 silencing increased the OCR in T24 and 5637 cells (Fig. 4j, Supplementary Fig. 4d). In contrast, overexpression of GLUT3 enhanced proliferation, invasion and glycolytic activity in bladder cancer cells (Fig. 4k–q). Thus, our results reveal that GLUT3 is crucial for the progression of bladder cancer.

### YTHDC1 suppresses the progression of bladder cancer partially through GLUT3

**Fig. 5:**
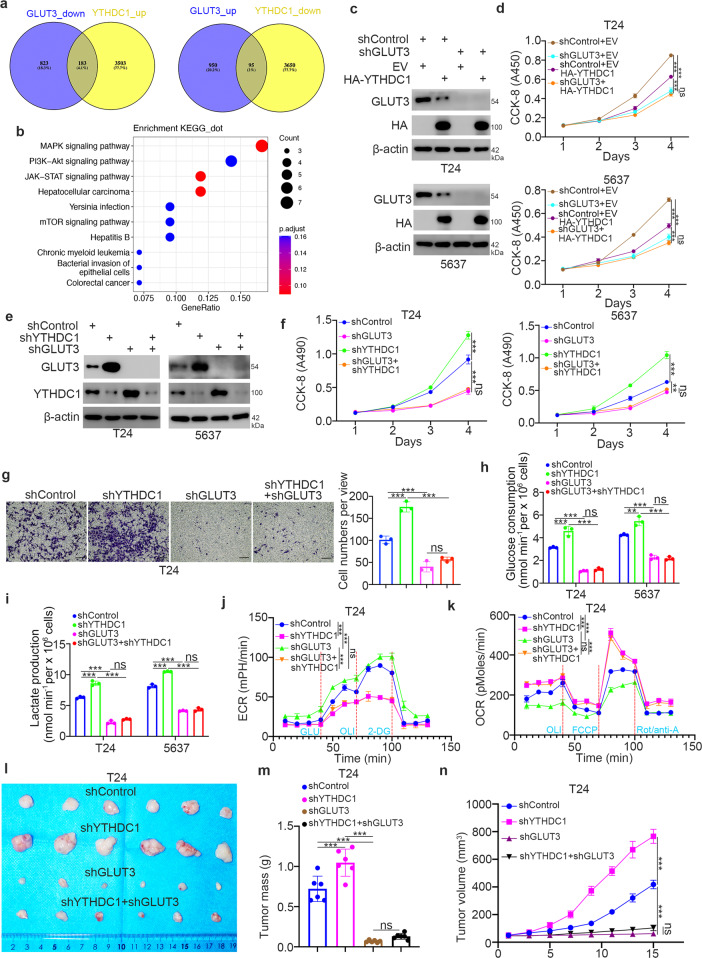
YTHDC1 inhibits the progression of bladder cancer partially through GLUT3. **a** and **b** Reanalysis of RNA-seq data related to YTHDC1 and GLUT3 knockdown to identify the common target genes (**a**). KEGG enrichment analysis was performed with these common target genes (**b**). **c** and **d** T24 and 5637 cells were transfected with the indicated constructs for 48 h. The cells were harvested for western blot analysis (**c**) and a CCK-8 assay (**d**). The data are presented as the mean ± SEM of three replicates. ns not significant; ***P < 0.001. **e–k** T24 and 5637 cells were transfected with the indicated constructs for 48 h. The cells were harvested for western blot analysis (**e**) and CCK-8 (**f**), transwell (**g**), glucose consumption (**h**), lactate production (**i**), ECR (**j**) and OCR (**k**) assays. The data are presented as the mean ± SEM of three replicates. ns, not significant; ***P < 0.001. **l–n** T24 cells were transfected with the indicated constructs for 72 h. After puromycin selection, cells were collected and subcutaneously injected into nude mice. Images of the excised tumors are shown in Panel **l**. The tumor masses and tumor growth curves are shown in Panels **m** and **n**. The data are presented as the mean ± SEM of six replicates. ns not significant; ***P < 0.001.

Since GLUT3 is the downstream gene of YTHDC1 in bladder cancer cells, we next determined whether GLUT3 is one of the key mediators of YTHDC1-induced suppression of bladder cancer. We reanalyzed the RNA-seq data related to YTHDC1 and GLUT3 modulation and found 183 (downregulated after GLUT3 knockdown and upregulated after YTHDC1 knockdown) and 95 (upregulated after GLUT3 knockdown and downregulated after YTHDC1 knockdown) genes (Fig. 5a). KEGG enrichment analysis of these 183 and 95 genes indicated that the MAPK, PI3K-AKT, JAK-STAT, hepatocellular carcinoma, and mTOR signaling pathways were the common pathways regulated by the YTHDC1-GLUT3 axis (Fig. 5b). Then, we generated T24 and 5637 cells with stable GLUT3 knockdown and simultaneous overexpression or knockdown of YTHDC1 (Fig. 5c and e). We showed that GLUT3 depletion diminished the inhibitory effects of YTHDC1 on growth, invasion, and the glycolytic process in T24 and 5637 cells (Fig. 5c–k, Supplementary Fig. 4e and f). Moreover, we established a nude mouse xenograft model to demonstrate that GLUT3 knockdown blocked tumor growth and that the combined knockdown of GLUT3 and YTHDC1 attenuated the promoting effect of YTHDC1 silencing on tumor growth (Fig. 5l–n). Taken together, these data suggest that YTHDC1 inhibits the malignant progression of bladder cancer cells via GLUT3.

### GLUT3 is involved in the ubiquitin-mediated proteolysis of YTHDC1 in bladder cancer cells

**Fig. 6:**
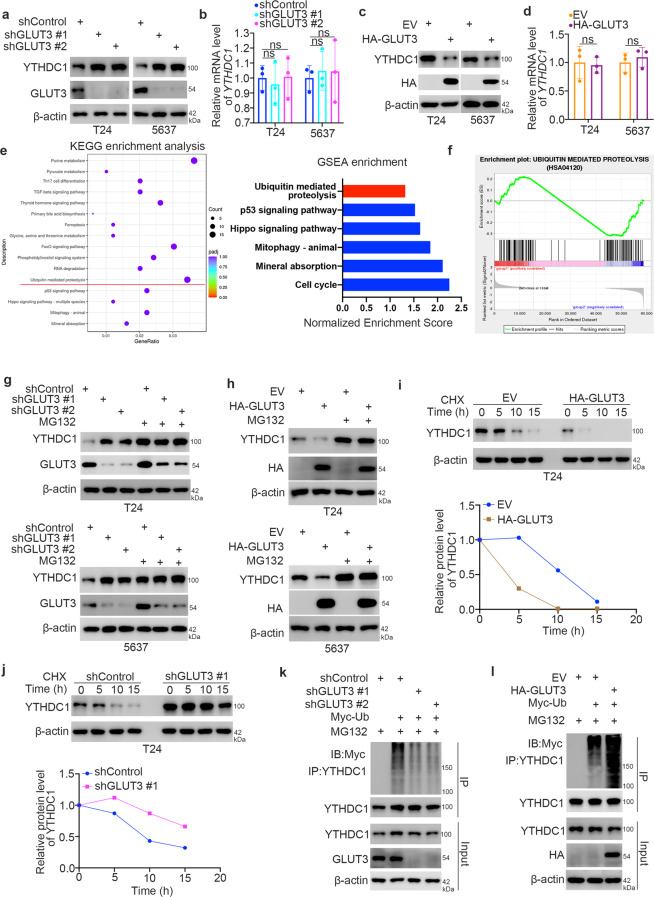
YTHDC1 degradation is induced by GLUT3 in bladder cancer cells. **a** and **b** T24 and 5637 cells were transfected with the indicated shRNAs for 48 h. The cells were harvested for western blot analysis (**a**) and RT‒qPCR (**b**). The data are presented as the mean ± SEM of three replicates. ns not significant. **c** and **d** T24 and 5637 cells were transfected with the indicated plasmids for 24 h. The cells were harvested for western blot analysis (**c**) and RT‒qPCR (**d**). The data are presented as the mean ± SEM of three replicates. ns not significant. **e** and **f** KEGG enrichment and GSEA of the RNA-seq data related to GLUT3 knockdown. **g** T24 and 5637 cells were transfected with the indicated shRNAs for 48 h. Then, the cells were treated with or without MG132 for 8 h before harvesting for western blot analysis. **h** T24 and 5637 cells were transfected with the indicated plasmids for 48 h. Then, the cells were treated with or without MG132 for 8 h before harvesting for western blot analysis. **i** T24 cells were transfected with the indicated plasmids for 48 h. The cells were treated with CHX and were collected for western blot analysis at different time points. **j** T24 cells were transfected with the indicated shRNAs for 48 h. The cells were treated with CHX and were collected for western blot analysis at different time points. **k** T24 cells were transfected with the indicated shRNAs and plasmids for 48 h. Then, these cells were treated with MG132 for another 8 h and subjected to IP and western blot analysis. **l** T24 cells were transfected with the indicated shRNAs and plasmids for 24 h. Then, these cells were treated with MG132 for another 8 h and subjected to IP and western blot analysis.

Interestingly, we found that GLUT3 knockdown seemed to increase the expression of YTHDC1 in T24 and 5637 cells (Fig. 5e). Consistent with this finding, the knockdown of GLUT3 increased the protein but not the mRNA level of YTHDC1 in T24 and 5637 cells (Fig. 6a and b). In contrast, overexpression of GLUT3 reduced the protein level but not the mRNA level of YTHDC1 in bladder cancer cells (Fig. 6c and d). Notably, KEGG enrichment analysis and GSEA of the RNA-seq data related to GLUT3 knockdown indicated that there was a close relationship between GLUT3 and ubiquitin-mediated proteolysis (Fig. 6e and f). Thus, we hypothesized that GLUT3 might regulate the protein stability of YTHDC1 in bladder cancer cells. To verify this, we treated T24 and 5637 cells with a 26 S proteasome inhibitor (MG132) after knockdown/overexpression of GLUT3 (Fig. 6g and h). We showed that MG132 treatment diminished the effect of GLUT3 on modulating the protein level of YTHDC1 in bladder cancer cells (Fig. 6g and h). Moreover, we demonstrated that overexpression of GLUT3 shortened but GLUT3 silencing prolonged the half-life of YTHDC1 in T24 cells (Fig. 6i and j). Furthermore, we found that GLUT3 knockdown decreased the polyubiquitination level of YTHDC1 in T24 cells (Fig. 6k). In contrast, GLUT3 overexpression increased the polyubiquitination level of YTHDC1 in cells (Fig. 6l). In summary, we showed that the protein stability of YTHDC1 is regulated by GLUT3 in bladder cancer cells.

### GLUT3 increases the expression of RNF183 to promote YTHDC1 degradation in bladder cancer cells

**Fig. 7:**
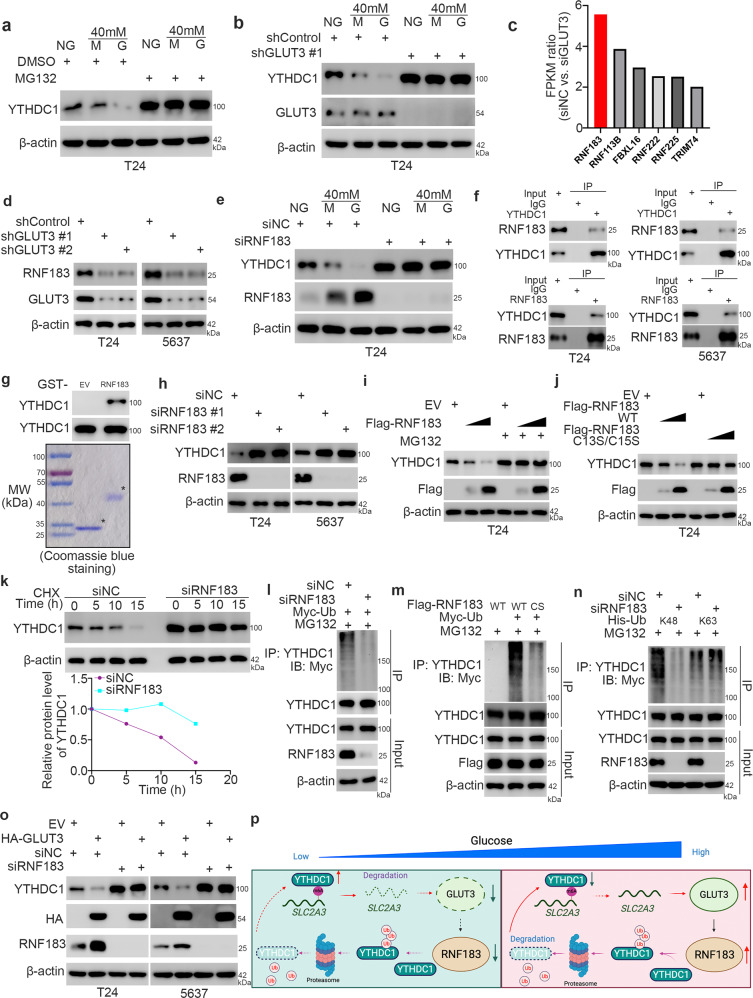
RNF183 is responsible for GLUT3 knockdown-induced YTHDC1 stabilization in bladder cancer cells. **a** T24 cells were treated with the indicated chemicals for 48 h. Then, the cells were harvested for western blot analysis. **b** T24 cells were transfected with the indicated shRNAs for 48 h. Then, the cells were treated with the indicated chemicals for another 48 h and subjected to western blot analysis. **c** RNA-seq analysis of the effects of GLUT3 knockdown indicated that knockdown of GLUT3 decreased the expression of many E3 ligases in 5637 cells. **d** T24 and 5637 cells were transfected with the indicated shRNAs for 48 h. The cells were harvested for western blot analysis. **e** T24 cells were transfected with the indicated siRNAs for 48 h. Then, the cells were treated with the indicated chemicals for another 48 h and subjected to western blot analysis. **f** Co-IP was performed in T24 and 5637 cells by using anti-RNF183 or anti-YTHDC1 antibodies. **g** A GST pulldown assay was performed with bacterially expressed GST-EV or GST-RNF183. **h** T24 and 5637 cells were transfected with the indicated siRNAs for 48 h. The cells were harvested for western blot analysis. **i** T24 cells were transfected with the indicated plasmids for 48 h. The cells were treated with or without MG132 for another 8 h and subjected to western blot analysis. **j** T24 cells were transfected with the indicated plasmids for 48 h. The cells were harvested for western blot analysis. **k** T24 cells were transfected with the indicated siRNAs for 48 h. The cells were treated with CHX and were collected for western blot analysis at different time points. **l** T24 cells were transfected with the indicated siRNAs and plasmids for 48 h. Then, these cells were treated with MG132 for another 8 h and subjected to IP and western blot analysis. **m** T24 cells were transfected with the indicated plasmids for 48 h. Then, these cells were treated with MG132 for another 8 h and subjected to IP and western blot analysis. **n** T24 cells were transfected with the indicated siRNAs and plasmids for 48 h. Then, these cells were treated with MG132 for another 8 h and subjected to IP and western blot analysis. **o** T24 and 5637 cells were transfected with the indicated constructs for 48 h. The cells were harvested for western blot analysis. **p** A model showing that YTHDC1 promotes SLC2A3 mRNA degradation to reduce the level of GLUT3 under low/normal glucose conditions. Hyperglycemia promotes YTHDC1 degradation by elevating the expression level of GLUT3/RNF183. Thus, a novel YTHDC1/GLUT3/RNF183 feedback loop that regulates disease progression and glucose metabolism was identified in bladder cancer cells.

The previous findings and our abovementioned results suggest that high glucose decreases the protein level but not the mRNA level of YTHDC1 in cells (Fig. 1a and Supplementary Fig. 1a). We sought to determine whether this process is mediated by the activation of proteasomal degradation. Interestingly, MG132 treatment blocked the decrease in the YTHDC1 protein level under high-glucose conditions (Fig. 7a). Then, we showed that GLUT3 knockdown attenuated the decrease in the YTHDC1 protein level under high-glucose conditions (Fig. 7b). Given that GLUT3 is not an E3 ligase, GLUT3 might indirectly promote the degradation of YTHDC1 in bladder cancer cells. It is worth noting that GLUT3 knockdown decreased the expression of six E3 ligases, as determined by analysis of GLUT3 knockdown-related RNA-seq data (Fig. 7c). Among these E3 ligases, RNF183 was the most downregulated (Fig. 7c). Then, the downregulation of RNF183 induced by GLUT3 knockdown was confirmed in both T24 and 5637 cells (Fig. 7d, Supplementary Fig. 5a). It has been well documented that RNF183, a member of the RING finger (RNF) protein family, is a hypertonicity-responsive ubiquitin ligase in cells^38^. We also found that hypertonic conditions slightly decreased the protein level of YTHDC1 in T24 cells (Fig. 1a). Surprisingly, the knockdown of RNF183 diminished the changes in the YTHDC1 protein level under hypertonic or high-glucose conditions (Fig. 7e). Thus, we hypothesized that RNF183 might be the bridge between GLUT3 and YTHDC1 degradation in bladder cancer cells. First, the results of coimmunoprecipitation (co-IP) and GST pulldown assays showed that RNF183 directly interacted with YTHDC1 (Fig. 7f and g). Then, RNF183 silencing by siRNA transfection was found to increase the protein but not the mRNA level of YTHDC1 in T24 and 5637 cells (Fig. 7h, Supplementary Fig. 5b). Overexpression of RNF183 reduced the protein level of YTHDC1 in T24 cells, and this effect was blocked by MG132 treatment (Fig. 7i). Moreover, we found that compared to wild-type RNF183, the RNF183 ligase activity-dead mutant (RNF183 C13S/C15S)^39^ could not reduce the protein level of YTHDC1 in T24 cells (Fig. 7j). Then, we demonstrated that knockdown of RNF183 prolonged the half-life and decreased the polyubiquitination of YTHDC1 in T24 cells (Fig. 7k and l). However, overexpression of wild-type RNF183 but not RNF183 C13S/C15S increased the polyubiquitination of YTHDC1 in T24 cells (Fig. 7m, Supplementary Fig. 5c). Furthermore, we showed that RNF183 led to K48-linked ubiquitination, which is the major ubiquitination event responsible for protein degradation^40,41^, of YTHDC1 in cells (Fig. 7n). Finally, we revealed that knockdown of RNF183 attenuated the decrease in the YTHDC1 protein level induced by GLUT3 overexpression in both T23 and 5637 cells (Fig. 7o). Taken together, these data suggest that the GLUT3/RNF183 axis promotes YTHDC1 degradation in bladder cancer cells (Fig. 7p).

## Discussion

Dysregulation of glucose metabolism is a characteristic of bladder cancer cells^42^. Aerobic glycolysis-dependent metabolism constitutes the major energy source for the proliferation of bladder cancer cells^42^. Thus, overexpression of genes related to glycolytic processes, such as GLUT1, GLUT3, HK2, LDHA, and PKM, is common in bladder cancer tissues and cells^42^. The GLUT family initiates the usage of glucose in cells^43^. Upregulation of GLUT family members has been found in various malignancies^43^. For example, GLUT3 is reported to be highly expressed in colorectal cancer and associated with an unfavorable prognosis in patients^44^. In addition, GLUT3 is specifically upregulated in triple-negative breast cancer (TNBC), which results in metastatic progression and is negatively correlated with the outcomes of TNBC patients^36^. GLUT3 enhances tumor cell growth by accelerating glucose uptake and fueling nucleotide synthesis^44^. In this study, we first showed that the downregulation of YTHDC1 increased the expression of GLUT3. Dai et al. also reported that low-glucose-related stress elevated the expression of GLUT3 through the AMPK/CREB1 pathway^44^. Since high-glucose conditions were found to decrease the expression of YTHDC1, our findings might provide another explanation for the regulation of GLUT3 by glucose stress. We performed RNA-seq analysis after GLUT3 knockdown to further explore the signaling pathways affected by GLUT3. We showed that many cancer-promoting pathways, including tumor angiogenesis, MAPK, and PI3K/AKT/mTOR pathways, were inactivated after GLUT3 silencing. Moreover, we demonstrated that GLUT3 can increase the expression of many E3 ligases. However, how these pathways or genes are regulated by GLUT3 is still unclear. The underlying mechanism needs to be studied in the future.

Although the molecular mechanism by which GLUT3 upregulates RNF183 is not clear, we demonstrated that RNF183 is the key mediator through which GLUT3 destabilizes YTHDC1 in bladder cancer cells. As an m^6^A reader, YTHDC1 regulates pre-mRNA splicing by enhancing SRSF3 expression but suppressing SRSF10 expression^33^. However, the role of YTHDC1 in malignant tumors is controversial. Recently, YTHDC1 was found to be elevated in acute myeloid leukemia (AML) and to be essential for the survival and development of AML cells^45^. Tang et al. mentioned that YTHDC1 facilitates the mRNA stability of SLC7A5 to promote the progression of colorectal cancer^46^. On the other hand, YTHDC1 was reported to impede the proliferation of glioma by downregulating the expression of VPS25^30^. Moreover, YTHDC1 was found to promote the maturation of miR-30d, which targets RUNX1 to suppress pancreatic tumorigenesis^47^. Moreover, the YTHDC1/miR-30d/RUNX1 axis also inhibits aerobic glycolysis by decreasing the expression of SLC2A and HK1^47^. Consistent with this finding, we showed that YTHDC1 inhibited the glycolytic process in bladder cancer cells by influencing the mRNA stability of GLUT3 in an m^6^A modification-dependent manner. The reason for the distinct function of YTHDC1 in different types of malignancies might be that m^6^A is one of the most common and abundant transcriptional modifications^19^, and the heterogeneity of tumors increases the complexity of the set of biological functions regulated by m^6^A modification^48^.

Both the above results and previous findings indicate that YTHDC1 is closely related to glucose metabolism in cells. Hyperglycemia was reported to downregulate the expression of YTHDC1 in keratinocytes^26^. YTHDC1 inhibits the glycolytic process through the miR-30d/RUNX1 axis in pancreatic cancer cells^47^. The findings of these two studies suggest a mutual interactive relationship between YTHDC1 and glucose metabolism. Here, we identified a positive feedback loop, namely, YTHDC1/GLUT3/RNF183, to clarify the relationship between YTHDC1 and glucose metabolism in bladder cancer cells.

Collectively, our results demonstrate that YTHDC1 is expressed at low levels in bladder cancer. Downregulation of YTHDC1 is negatively correlated with the outcome of patients with bladder cancer. We also showed that YTHDC1 suppresses the malignant progression of and the glycolytic process in bladder cancer cells in an m^6^A-dependent manner and determined that this effect is partially mediated by GLUT3. Moreover, GLUT3 was found to destabilize YTHDC1 by upregulating RNF183 expression. Therefore, we identified a novel YTHDC1/GLUT3/RNF183 feedback loop that regulates disease progression and glucose metabolism in bladder cancer. This study provides new insight regarding the pathogenesis of bladder cancer under hyperglycemic conditions and might reveal ideal candidates for the development of drugs for bladder cancer.

## Supplementary information

Supplementary information

## Data Availability

The datasets used and/or analyzed during the current study are available from the corresponding authors on reasonable request.
