# Supplementary material for: The YTHDC1/GLUT3/RNF183 axis forms a positive feedback loop that modulates glucose metabolism and bladder cancer progression
Source: Exp Mol Med. 2023 Jun 1;55(6):1145–58. doi: 10.1038/s12276-023-00997-z (PMC10318083; doi:10.1038/s12276-023-00997-z)
Supplement: Supplementary file 1 — Supplementary information [file 12276_2023_997_MOESM1_ESM.pdf]

The YTHDC1/GLUT3/RNF183 axis forms a positive feedback loop that modulates glucose metabolism and bladder cancer progression

Bin Yan, Xurui Li, Mou Peng, Yali Zuo, Yinhuai Wang, Pian Liu, Weigang Ren, Xin Jin

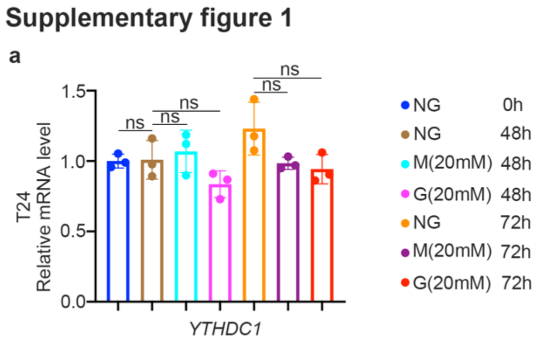

Supplementary Fig. 1.

a, T24 cells were treated with the indicated chemicals for 48 h or 72 h. Then, cells were harvested for RT-qPCR analysis. Data presents as mean ± SEM with three replicates. ns, not significant; **, $P < 0.01$; ***, $P < 0.001$.

Supplementary figure 2

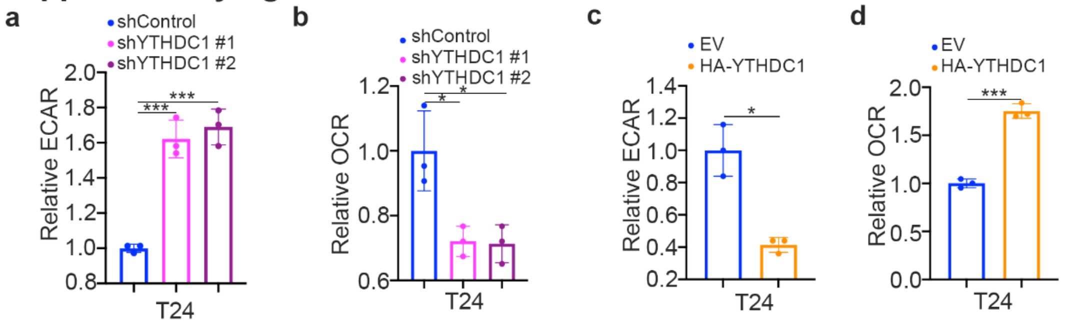

Supplementary Fig. 2.

a and b, T24 cells were transfected with the indicated shRNAs for 48 h. Cells were harvested for ECAR and OCR assay. Data presents as mean ± SEM with three replicates. *, P < 0.05; ***, P < 0.001. **c and d**, T24 cell cells were transfected with the indicated plasmids for 24 h. Cells were harvested for ECAR and OCR assay. Data presents as mean ± SEM with three replicates. *, P < 0.05; ***, P < 0.001.

Supplementary figure 3

a

Stacked peak regions of YTHDC1 on SLC2A3:

Show 10 entries Search:

SiteID	StackedRegion	BindTranscriptNum	ClipExpNum
YTHDC1:CH000011832	chr12:8073814–8073830[–]	1	3
YTHDC1:CH000011833	chr12:8073896–8073907[–]	1	2
YTHDC1:CH000011834	chr12:8074160–8074184[–]	1	2

b

chr12	8073944	8073945	-	m6A_site_92521	m6A	294.79	71	SLC2A3,SLC2A3	retained_intron,protein_coding	exon,utr3
chr12	8073929	8073930	-	m6A_site_92520	m6A	299.78	71	SLC2A3,SLC2A3	retained_intron,protein_coding	exon,utr3
chr12	8073812	8073813	-	m6A_site_92519	m6A	371.87	63	SLC2A3,SLC2A3	retained_intron,protein_coding	exon,utr3

c

Detection region 1 (DC1): 8073800-8073899

Detection region 2 (DC2): 8073900-8073999

d

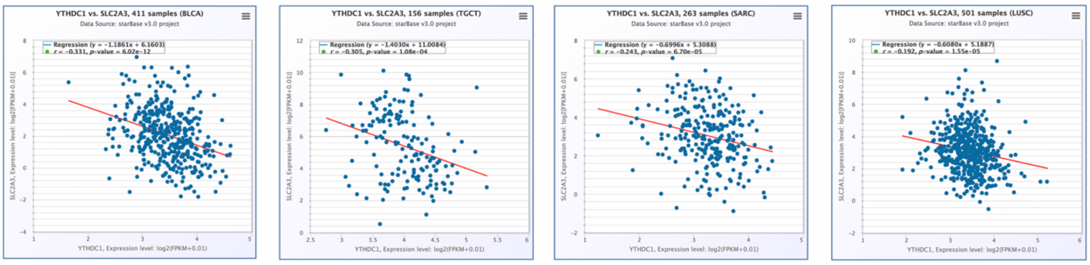

e Gene: SLC2A3 ENSG00000059804

Show/hide columns (1 hidden)

Name	Transcript ID	bp	Protein	Biotype	CCDS	UniProt	RefSeq Match	Flags
SLC2A3-001	ENST00000075120.7	3915	496aa	Protein coding	CCDS8586	P11169	-	GENCODE basic
SLC2A3-010	ENST00000544291.1	577	141aa	Protein coding	-	F5GYR5	-	CDS 3' incomplete
SLC2A3-009	ENST00000544936.1	589	48aa	Nonsense mediated decay	-	F5GYX0	-	-
SLC2A3-013	ENST00000543435.1	383	No protein	Processed transcript	-	-	-	-
SLC2A3-003	ENST00000486749.1	4414	No protein	Retained intron	-	-	-	-
SLC2A3-002	ENST00000495813.1	1784	No protein	Retained intron	-	-	-	-
SLC2A3-006	ENST00000469295.1	1030	No protein	Retained intron	-	-	-	-
SLC2A3-005	ENST00000490763.1	943	No protein	Retained intron	-	-	-	-
SLC2A3-012	ENST00000479059.3	870	No protein	Retained intron	-	-	-	-
SLC2A3-004	ENST00000476634.1	763	No protein	Retained intron	-	-	-	-
SLC2A3-011	ENST00000541671.1	590	No protein	Retained intron	-	-	-	-

f

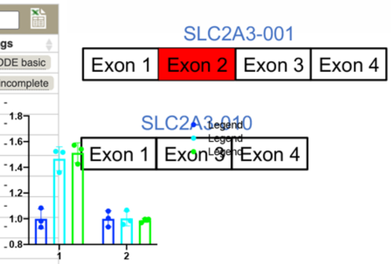

g

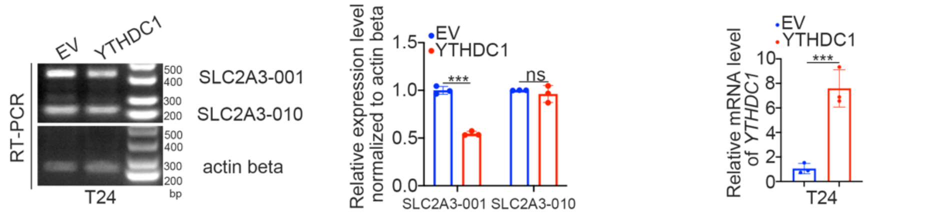

h

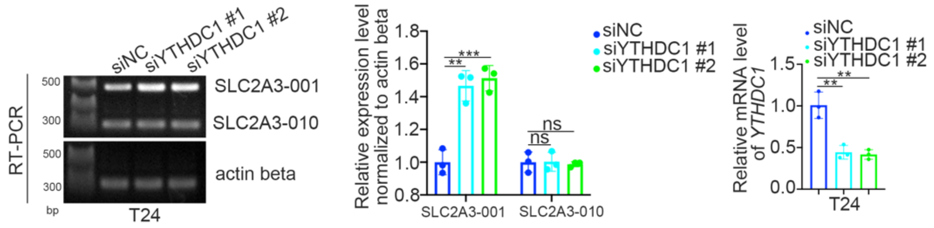

Supplementary Fig. 3.

a, The ENCORI web tool showed the binding site of YTHDC1 on SLC2A3. **b,** the RMBase v2.0 web tool showed the m^6A modification site of SLC2A3. **c**, the m^6A detection region on SLC2A3. **d**, The ENCORI web tool was used to detect the correlation between SLC2A3 and YTHDC1 in bladder cancer (BLCA), tenosynovial giant cell tumor (TGCT), sarcoma (SARC), and Lung squamous cell carcinoma (LUSC). **e**, the Ensembl dataset (https://grch37-archive.ensembl.org/) was used to explore the splicing isoform of SLC2A3. **f**, the model depicting the SLC2A3 full length (SLC2A3-001) and the splicing isoform (SLC2A3-010). **g**，T24 cells were transfected indicated plasmids for 24 h. Cells were harvested for RT-PCR and RT-qPCR analysis. The two isoforms of SLC2A3 were quantified by using the Image J software and normalizing to the amount of action beta. Data presents as mean ± SEM with three replicates. Ns, not significant; ***, P < 0.001. **h**, T24 cells were transfected with indicated siRNAs for 48 h. Cells were collected for RT-PCR and RT-qPCR analysis. The two isoforms of SLC2A3 were quantified by using the Image J software and normalizing to the amount of action beta. Data presents as mean ± SEM with three replicates. Ns, not significant; **, P < 0.01; ***, P < 0.001.

Supplementary figure 4

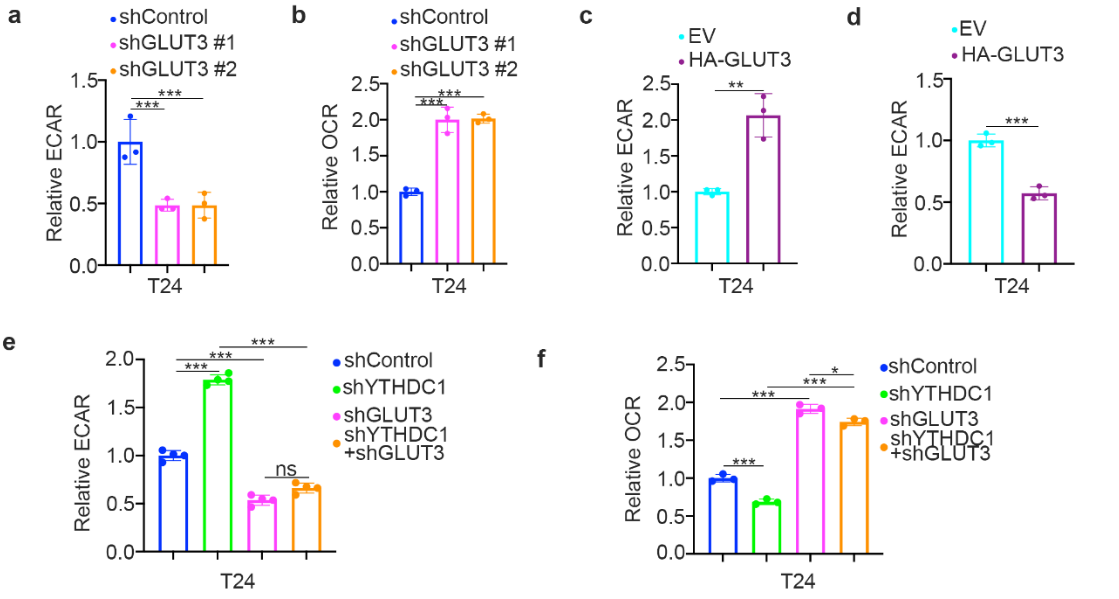

Supplementary Fig. 4.

a and b, T24 cells were transfected with the indicated shRNAs for 48 h. Cells were harvested for ECAR and OCR assay. Data presents as mean ± SEM with three replicates. *, P < 0.05; ***, P < 0.001. **c and d**, T24 cell cells were transfected with the indicated plasmids for 24 h. Cells were harvested for ECAR and OCR assay. Data presents as mean ± SEM with three replicates. *, P < 0.05; ***, P < 0.001. **e and f**, T24 cells were transfected with the indicated shRNAs for 48 h. Cells were harvested for ECAR and OCR assay. Data presents as mean ± SEM with three replicates. Ns, not significant; *, P < 0.05; ***, P < 0.001.

Supplementary figure 5

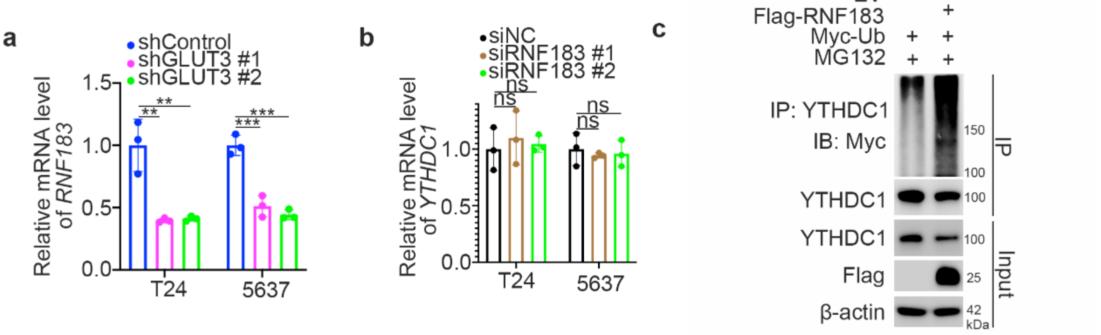

Supplementary Fig. 5.

a, T24 and 5637 cells were transfected with the indicated shRNAs for 48 h. Cells were harvested for RT-qPCR analysis. Data presents as mean ± SEM with three replicates. **, P < 0.01; ***, P < 0.001. **b**, T24 and 5637 cells were transfected with the indicated plasmids for 48 h. Cells were harvested for RT-qPCR analysis. Data presents as mean ± SEM with three replicates. Ns, not significant. **c**, T24 cells were transfected with the indicated plasmids for 48 h. Then, these cells were treated with MG132 for another 8 h and subjected to IP and western blot analysis.

Supplementary Material and Methods

Cell migration and invasion assay

For migration assay, the cell layer in 6-well plate was scratched and detached cells were removed. For each sample, at least three scratched fields were photographed immediately. The BioCoat Matrigel invasion chamber (BD Biosciences) was used to evaluate the cell invasion ability according to the protocol of the manufacturer. Cells were cultured in the insert for 24 h. Cells were fixed in methanol for 15 min and then stained with 1 mg/ml crystal violet for 20 min. At least three fields for each group were photographed after staining, and invaded cells were counted.

In vitro angiogenesis assay

Approximately 1×10^5 HUVEC cells were resuspended with tumor-conditioned supernatant in each well of a 24-well plate containing 250 µl Matrigel (BD Bioscience, USA). The supernatant of bladder cancer cells was added to HUVEC cells. HUVEC cells were cultured and monitored for tube formation up to 12 h.

Glucose consumption and lactate production assay

Bladder cancer cells (1×10^5) were seeded in 6-well plates and cultured in DMEM medium without phenol red (Invitrogen, USA). The spent medium was collected and measured by a glucose assay kit (# ab65333, Abcam, China). Glucose consumption was calculated by subtracting the difference in glucose concentrations between the

spent medium and the unused medium. Lactate levels were measured using a lactate assay kit (#ab65330, Abcam, China).

Extracellular acidification rate and oxygen consumption rate

The extracellular acidification rate (ECAR) and oxygen consumption rates (OCR) of bladder cancer cells were measured by XF24 analyzer (Seahorse Bioscience). After calibration of the analyzer, sequential injection of glucose, oligomycin A and 2-DG were used to detect the extracellular acidification rate and sequential injection of oligomycin A, FCCP, antimycin A and rotenone were used to detect the oxygen consumption rate.

Bladder cancer cells transfected with indicated constructs were seed into the 96 well plate with 5×10^4 per well. The next day, cells were cultured in the incubator at 37 °C with CO_2 free for three hours. Then, cells were subjected to measure the extracellular acidification rate (ECAR or ECR) and oxygen consumption rates (OCR) by using the Extracellular acidification assay kit (# ab197244, Abcam) and Extracellular Oxygen Consumption kit (# ab197272, Abcam) following the manufacture's instruction. The ECAR values were collected at 60 min by micro-plate reader system using excitation and emission wavelengths of 380 and 620 nm. The OCR values were collected at 60 min by micro-plate reader system using excitation and emission wavelengths of 380 and 650 nm.

GSEA for the key gene

GSEA: KIRC patients were first divided into two groups according to the median expression level of the key gene. Then, differential expression analysis was applied between the high and low expression groups. Input genes for GSEA were sorted by their logFC values. Signaling pathways activated or suppressed by the key gene were decided by the normalized enrichment score (NES) value derived from GSEA.

ssGSEA: ssGSEA was used to calculate separate enrichment scores for each pairing of a KIRC sample and KEGG gene set. The ssGSEA score was further rescaled by min-max normalization method. Correlation analysis was performed between expression values of key gene and NES of signaling pathways.

Supplementary Table 1. The siRNA and shRNA sequences.

siYTHDC1 #1	5'- GAGAAAGATGGAGAACTTAATGT-3'
siYTHDC1 #2	5'- AACAAGATAAAAATGAGAAAAAG-3'
siYTHDC1 #3	5'- TGGATCAGATGAGAAAAAGAAGG-3'
siRNF183 #1	5'- CCACCATGTCATCCTGGAAGG-3'
siRNF183 #2	5'- TGCTCATATTCTCCATCTTTTGG-3'
shGLUT3#1	5'-CACCGAGAAGATCATAAAGGAATTTATCTCGAGATAAATTCCTTTATGATCTTCTC-3'
shGLUT3 #2	5'-CACCGTCCCAGATTTTTGCTCATTAACACTCGAGTGTTAATGAGCAAAAATCTGGGA-3'
shYTHDC1 #1	5'-CACCAGGAGAAAGATGGAGAACTTAATCTCGAGATTAAGTTCTCCATCTTTCTCC-3'
shYTHDC1 #2	5'-CACCATCGGAAAATTCGTCTATCAAGTCTCGAGACTTGATAGACGAATTTTCCGA-3'

Supplementary Table 2. The primer sequences for RT-qPCR.

Gene （Human）	Forward primer (5′ - 3′)	Reverse primer (5′ - 3′)
actin beta	ACAGAGCCTCGCCTTTGCC	TGGCCATCTCTTGCTCGAAG
YTHDC1	GGAGGGCCAAATCTCCTACG	CTTTTCGGACAGCACGAACG
SLC2A3	TCCCTTTCCCTTCTTGGTGC	GTGAGAAATGGGACCCTGCC
RNF183	AAGGGGAAGAGGCTCTGGAA	ATGGCCTGAACAATCCCTGG

Supplementary Table 3. The primer for RIP-PCR

Gene （Human）	Forward primer (5′ - 3′)	Reverse primer (5′ - 3′)
SLC2A3 （YTHDC1）	CAGAAGAGGATGTCCAGGAA	CTCCCTCAACAAGGGAGAGA
SLC2A3 (For splicing variants)	ATGGGGACACAGAAG	CTGGGCCACCAGAATTCCAACAA
actin beta (For splicing variants)	CTCGCCTTTGCCGATCC	GGGGTACTTCAGGGTGAGGA

Supplementary Table 4. The primer for MeRIP-qPCR

Gene （Human）	Forward primer (5′ - 3′)	Reverse primer (5′ - 3′)
DC1	CCAATCCCTCCTGAAATGAA	CTGCGGTTTGTTGGATCTTC
DC2	AGAAGCGTCCTGGGTTCAT	GCTAAGGAGACCACCACCAA
